# A novel technique for the per-anal extraction of spherical rectal foreign bodies

**DOI:** 10.1308/003588412X13373405386015b

**Published:** 2012-09

**Authors:** RJ Codd, TJ Havard

**Affiliations:** Royal Glamorgan Hospital, Llantrisant,UK

## BACKGROUND

It is desirable to remove rectal foreign bodies per-anally to avoid laparotomy and possible colostomy.^1,2^ Spherical objects can pose a specific problem due to difficulty in gaining purchase digitally or with an instrument. Obstetric forceps have been used previously^3–5^ but their positioning and extraction can be technically difficult and potentially traumatic. We present a technique whereby a single blade of a pair of Neville-Barnes forceps can be used easily to extract spherical objects.

## TECHNIQUE

Under general anaesthesia with the patient positioned in lithotomy, the assistant should apply downward suprapubic pressure to move the object caudally. Following a digital rectal examination to identify the object’s position, a well lubricated blade of the forceps is inserted using the left hand. The operator’s right index finger is used to guide the blade over the anterior surface of the object. Once in position, the blade is used to apply pressure through the object so it comes into contact with the curved anterior surface of the sacrum. It is then removed slowly, maintaining pressure through the object, thus allowing extraction.

## DISCUSSION

The use of a single obstetric blade in this way has not been described previously. It is a useful technique that uses the normal anatomy of the pelvis to the surgeon’s advantage. Compared to a pair of obstetric forceps, a single blade can be positioned easily and should cause less trauma to the anal canal. This technique was used to extract a 7cm rubber ball from the mid-rectum, thus avoiding laparotomy and potential stoma.
